# Forecasting the daily demand for emergency medical ambulances in England and Wales: a benchmark model and external validation

**DOI:** 10.1186/s12911-023-02218-z

**Published:** 2023-07-11

**Authors:** Thomas Monks, Alison Harper, Michael Allen, Lucy Collins, Andrew Mayne

**Affiliations:** 1 University of Exeter Medical School, University of Exeter, Heavitree Road, Devon EX1 2LU Exeter, UK; 2 NIHR Applied Research Collaboration Wessex, University of Exeter, Heavitree Road, Exeter, EX1 2LU Devon UK; 3 Data Science Team, South Western Ambulance Service NHS Foundation Trust, Eagle Way, Exeter, EX2 7HY Devon UK; 4 Service Evaluation & Improvement Team, Somerset NHS Foundation Trust, Musgrove Park Hospital, Taunton, TA1 5DA Somerset UK

**Keywords:** Emergency ambulance, Forecasting, External validation

## Abstract

**Background:**

We aimed to select and externally validate a benchmark method for emergency ambulance services to use to forecast the daily number of calls that result in the dispatch of one or more ambulances.

**Methods:**

The study was conducted using standard methods known to the UK’s NHS to aid implementation in practice. We selected our benchmark model from a naive benchmark and 14 standard forecasting methods. Mean absolute scaled error and 80 and 95% prediction interval coverage over a 84 day horizon were evaluated using time series cross validation across eight time series from the South West of England. External validation was conducted by time series cross validation across 13 time series from London, Yorkshire and Welsh Ambulance Services.

**Results:**

A model combining a simple average of Facebook’s prophet and regression with ARIMA errors (1, 1, 3)(1, 0, 1, 7) was selected. Benchmark MASE, 80 and 95% prediction intervals were 0.68 (95% CI 0.67 - 0.69), 0.847 (95% CI 0.843 - 0.851), and 0.965 (95% CI 0.949 - 0.977), respectively. Performance in the validation set was within expected ranges for MASE, 0.73 (95% CI 0.72 - 0.74) 80% coverage (0.833; 95% CI 0.828-0.838), and 95% coverage (0.965; 95% CI 0.963-0.967).

**Conclusions:**

We provide a robust externally validated benchmark for future ambulance demand forecasting studies to improve on. Our benchmark forecasting model is high quality and usable by ambulance services. We provide a simple python framework to aid its implementation in practice. The results of this study were implemented in the South West of England.

**Supplementary Information:**

The online version contains supplementary material available at 10.1186/s12911-023-02218-z.

## Introduction

Ambulance response times can be critical to patient outcomes for serious clinical events such as cardiac arrest [1], stroke [2], and major trauma [3]. Managing ambulance provision efficiently is therefore critically important to health outcomes. Part of that management is having an accurate forecast of expected demand that can be used to plan and schedule appropriate workforce at the regional and daily level. Forecasting studies of demand for emergency medical services (EMS) date back over three decades [4–12]. In this time, there has been some incremental improvement in methods most notably in developing spatial temporal forecasting methodology using neural network architectures [6, 11, 12]. The study by Martin et al. [12] also demonstrated that standard time series forecasting methods provide comparable prediction accuracy to machine learning methodology. The promise shown in these studies is yet to transfer to wide-scale implementation in ambulance services. We argue that this stems from a number of limitations of the current evidence. A fundamental weakness is that studies are single site with no external validation of the forecasting methods chosen. Of these studies few have used a scale independent measure of forecast accuracy [11, 12]. It is therefore difficult to robustly compare forecast accuracy across existing studies and settings. In effect, the current evidence makes it difficult for an EMS or researcher to judge if their forecasting methods are on par, fall below or exceed a state-of-the-art benchmark. There is one additional subtle limitation to the evidence. Existing studies have tended to focus evaluation on point forecast accuracy; for example, the accuracy of a prediction of demand next Tuesday. In a statistical perspective on forecasting, a point forecast is accompanied by a prediction interval: a range of values that a future value might take with a high probability [13]. For example, next Tuesday’s prediction could be supplied with a 95% prediction interval stating that the actual value for next Tuesday should lie within a given range with probability 0.95. Achieving adequate prediction interval coverage is difficult and an understanding of a chosen method’s capability is of high importance.

This study aims to provide EMS forecasting benchmarks for future research to incrementally improve on. To aid EMS workforce planning, we predict the daily number of calls that result in the dispatch of one or more ambulances [5]. Our objectives are to establish statistical benchmarks for point forecast accuracy and prediction interval coverage up to 12 weeks (84 days) ahead. We select the most accurate forecasting method from 14 established methods and present details of the forecast error distribution at 7 day intervals. We then externally test the selected method in three further ambulance trusts (in the UK an ambulance trust provides services across a region). Overall we test the method in 21 time series. Finally, we also provide an easy to use MIT licensed (free and open) Python framework for deploying the model. Our results enable future replication studies or studies of new methods to directly compare their accuracy to our benchmark. EMS data science teams developing in-house forecasting tools or purchasing commercial forecasting systems can also compare results and spend NHS resource wisely.

## Methods

We build multiple time series forecasting models using established methods. Model selection is conducted by time series cross validation evaluating both point forecast accuracy and prediction interval coverage. All models are compared to a naive statistical baseline model. We then test the selected method in a simulated forecasting setting (seven regions within a NHS Trust). The accuracy of the method in our test set is our benchmark. We test the external validation of the benchmark by applying the method to a further 13 regions from three different ambulance trusts in the United Kingdom.

### Study setting

We develop our benchmark model using data from the South Western Ambulance Service NHS Foundation Trust (SWASFT). SWASFT is an NHS Trust in England that provides emergency medical services to a population of 5.6 million spread over a mixed urban/rural region of 26,000 km^2^. The service receives an average 2,300 calls per day that require the dispatch of one or more ambulances. Forecasts are rerun every week and are used to set the staffing rotas three months ahead. The South West region can be broken down into seven sub-regions / time series: Devon, Cornwall, Dorset, Somerset, Gloucestershire, Wiltshire, and ‘Bristol, North Somerset and South Gloucestershire (BNSSG)’.

Our external validation is comprised of data from London Ambulance Service (LAS; 5 time series; population 8.6m; area size 1,570km^2^), the Welsh Ambulance Service Trust (WAST; 4 time series; pop 3m, area 20,740km^2^) and the Yorkshire Ambulance Service (YAS; 6 time series; pop 5m; area 15,540km^2^). Overall the trusts serve a population of over 20 million people in the UK.

### Outcome measures

We measure both point forecast error and prediction interval coverage of the forecasts. For point forecast error, our main outcome measure is the *Mean Absolute Scaled Error (MASE)* as this provides an easy to understand relative error measure that can be compared across ambulance trusts. For MASE, we scale the out of sample *mean absolute error* by the equivalent one-step within-sample error from a Seasonal Naive model [14]. We also report two secondary point forecast error measures. A second relative error measure is the *symmetric Mean Absolute Percentage Error (sMAPE)*. As it has been used elsewhere [5], we also provide a scale-dependent measure via the *Root Mean Absolute Squared Error (RMSE)*, although this can only be used in the context of the specific time series. Prediction interval coverage measures the proportion of out-of-sample observations that fall within a prediction interval with an expected probability. For example, it is expected that 80% of points will fall within an 80% prediction interval. For cross-validation, we report the 80% and 95% prediction interval coverage. We report the full forecast distribution for the final benchmark model.

### Data sources

The study was conducted at a daily time-series level. Each observation represents the daily count of emergency calls that resulted in the dispatch of one or more emergency ambulances. Each days calls are logged by the ambulance provider. The data used here are a subset of this total that result in an ambulance dispatch. For model development, each sub-region in the data were broken into training (01/01/13 - 30/06/17; $n = 1279$), validation (01/07/17 - 31/01/18; $n = 549$) and test sets (01/01/2019 - 31/12/2019; $n = 365$). All data available on record was used. The validation period differs from test in that it is used to tune and select models. Data were validated by combining independent screening of time series for anomalies and through NHS data checks before release. Researchers had no access to individual patient level data.

### Analysis environment

All analysis code was written in Python 3.7.5 and R 3.6.1. Python forecasting libraries used were pmdarima v1.5.3 [15], fbprophet v0.5 [16], statsmodels v0.11.1 [17], tbats v.1.0.10 [18], forecast-tools v0.1.5 [19] and from R we used Rssa v1.0.2 [20]. Data cleaning and manipulation were done using Pandas [21] and NumPy [22]. All charts were produced with MatPlotLib [23]. to enable the results of the benchmark study to be reproduced we followed the Turing Way [24]. We provide a docker image containing the exact software, code and data used (https://hub.docker.com/r/tommonks01/swast-benchmark/). Instructions to use the docker image, data and analysis code are available online [25] (https://github.com/TomMonks/swast-benchmarking). The computational analyses were run on Intel i9-9900K CPU with 64GB RAM running the Pop!_OS 20.04 Linux. Our benchmark model has been released as a MIT licensed (free and open) Python package [26]. A cloud runnable tutorial, via BinderHub, is available from https://github.com/TomMonks/swast-forecast-tool.

### Candidate models

We evaluated 14 candidate forecasting methods, listed in Table 1, relative to a naive benchmark. We selected established candidate methods (many of which are known to the UK National Health Service); for example, Holt-Winters Exponential Smoothing, Autoregressive Integrated Moving Average (ARIMA), Harmonic regression (regression with ARIMA errors using fourier series exogenous variables to represent seasonality), TBATS (Trigonometric seasonality, Box-Cox transformation, ARMA errors, Trend and Seasonal components) [27], and Singular Spectrum Analysis (SSA). We also included four ensembles (an unweighted average) of methods: three of individual methods already included as well as a standard ensemble *comb* [28].

We use a modern approach to ARIMA model selection by automatic selection of the model using the Hyndman-Khandakar algorithm. The number of fourier terms in harmonic regression were selected by minimising Akaike Information Criterion. The most modern method we employ is Facebook Prophet which was designed to handle higher frequency data that may have multiple periodicity. Prophet is similar to a Generalised Additive Model in that it is a curve fitting approach. All methods are simple for ambulance services to implement and available in either Python or R.

**Table 1:** Candidate forecasting methods

Method	Description
1	Holt-Winters Exponential Smoothing
2	Automatic Autoregressive Integrated Moving Average (autoARIMA)
3	Lagged regression (autoregression) with holidays and seasonal indexes
4	Lagged regularised regression (elastic-net) with seasonal indexes
5	Regression with holidays and ARIMA errors
6	Regression with holidays, seasonal indexes and ARIMA errors
7	Harmonic Regression (fourier terms) with holidays
8	Singular Spectrum Analysis
9	Facebook Prophet
10	Trigonometric seasonality, Box-Cox transformation (TBATS)
11	Comb: Simple Exponential Smoothing, Linear Trend, damped trend
12	Ensemble of [1] and [2]
13	Ensemble of [7], [5]
14	Ensemble of [1], [9] and [5]

### Statistical analysis

The study consisted of four stages. Table 2 summarises the statistical procedure and data used to select a benchmark method and perform an external evaluation.

**Table 2:** Statistical analysis procedure

Stage	TSCV methods	Data used
1. Screening	Seasonal naive benchmark	Trust level series
	10 X individual standard methods	01/01/13 - 31/12/2018
	5 x combination forecasters	
2. Elite screening	Top 2 methods from stage 1	7 regional series
		01/01/13 - 31/12/2018
3. Simulated forecast setting	Selected method	7 regional series
		Train: 01/01/13 - 31/12/2018
		Test: 01/01/2019 - 31/12/2019
4. External evaluation	Selected method	13 external series
		Train: 01/01/10 - 31/12/2018
		Test: 01/01/2019 - 31/12/2019

#### Stage 1: screening

A naive baseline forecasting method was chosen. This was to ensure that the sophisticated methods we test in the study were only considered for the final benchmark if they provided more accurate point forecasts than the simplest of models. As emergency care demand data are seasonal we opted for the well-known Seasonal Naive method [13]. This method works by using the most recent observation for the same day and carrying it forward. For example, if we are forecasting next Tuesday then the observation from the most recent Tuesday is used as the predicted value.

The large list of methods were initially screened using a method of time series cross-validation called rolling forecast origin [29]. To avoid leakage of future observations, the method incrementally moves the forecast origin forward in time and then makes a prediction. For each new fold we implemented a stride of seven days. We performed a two stage model selection procedure. In this first stage, we used an aggregate regional level time series to screen and identify the most promising candidate models for up to a 365 day forecast (27 folds).

#### Stage 2: elite screening

In the second stage, our top two ‘elite’ methods, in relation to our chosen outcomes and seasonal naive benchmark, were compared using seven sub-regional time series. For the 84 day horizon we had sufficient data to produce 67 validation folds for each sub-region.

#### Stage 3: simulated forecast setting

Our test set provided 365 observations for each of the seven sub-regions. This enabled us to make 41 simulated forecasts of 7 to 84 days. Our benchmark therefore includes a range of MASE and coverage metrics that might be expected in practice.

#### Stage 4: external validation

For external validation, we repeated the simulated forecast procedure within 13 test sets for London (5 01/01/10 - 31/12/2019), Yorkshire (01/01/13 - 31/12/2019) and Wales (01/10/15 - 31/12/2019). We report how the selected model compared across MASE and coverage metrics observed to those expected in our benchmark analysis.

## Results

### Training data

**Fig. 1:**
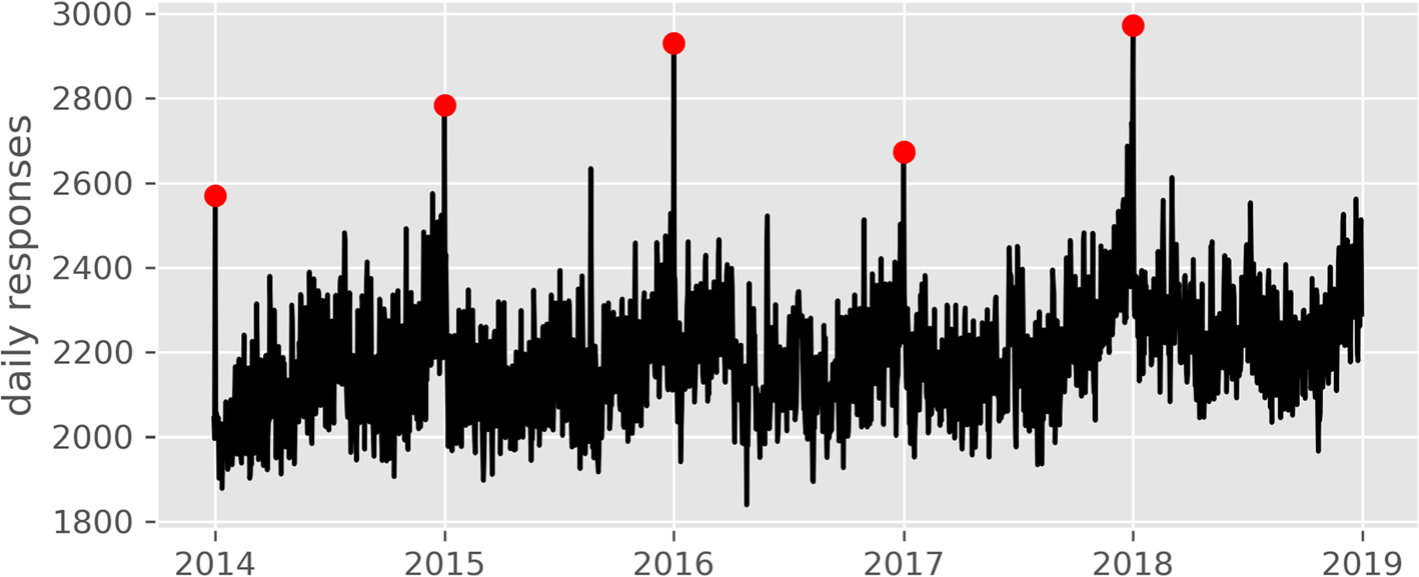
Time series of training data. Trust level daily number of calls that require one or more ambulance dispatches. Extreme observations observed on new years day marked with red dot

**Fig. 2:**
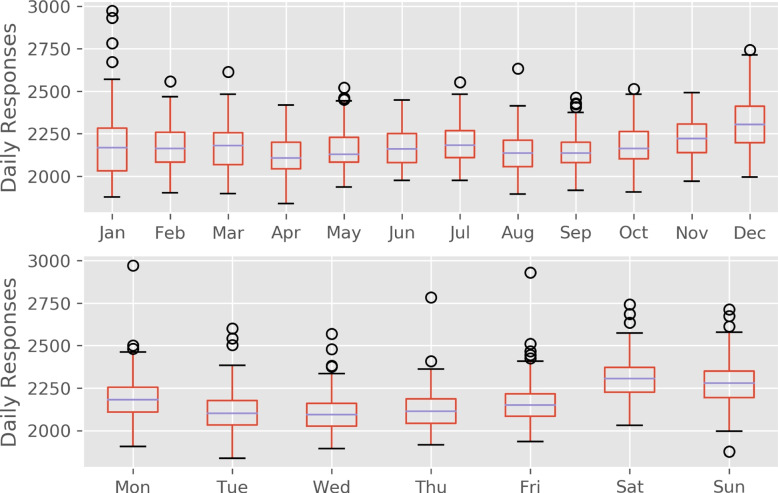
Annual and weekly seasonality. Top figure illustrates the variation in demand by month of the year. Bottom figure illustrates variation by day of week

There were no missing observations in the training data. Figure 1 depicts the training data time series. The median number of calls requiring an ambulance dispatch per day was 2169 (IQR 2083 - 2269). Extreme days (exceeding the 99th percentile) are observed on the 1st January every year (New Year’s day) where the median number of calls increased to 2783 (IQR 2673 - 2930). The data displayed a slight upward trend over time rising from median per day of 2135 in 2014 to 2257 by 2018. There was variation in demand by month of year and day of the week (Fig. 2).

### Naive benchmark

The magnitude of the point forecasts of the seasonal naive method increased with the forecast horizon (see Table 3). On average, the naive method achieved a MASE of 0.94 (0.35) over 7 days. For the trust, this represents a sMAPE and RMSE of 3.5% and 96.9 calls respectively. Over an 84 day horizon MASE increased to 1.34, (a 30% increase). By 365 days MASE had increased to 1.49 (0.46).

**Table 3:** Cross-Validation of Seasonal Naive Point Forecasts

Horizon (days)	MASE	sMAPE	RMSE
7	0.94 (0.35)	3.48 (1.19)	96.91 (37.11)
14	1.06 (0.39)	3.94 (1.32)	109.71 (41.88)
21	1.12 (0.41)	4.12 (1.38)	115.38 (42.94)
28	1.14 (0.40)	4.19 (1.33)	118.34 (42.92)
35	1.18 (0.40)	4.35 (1.34)	123.26 (42.33)
42	1.23 (0.40)	4.51 (1.33)	128.11 (40.34)
49	1.25 (0.39)	4.61 (1.32)	131.40 (39.79)
56	1.27 (0.39)	4.67 (1.33)	133.95 (40.20)
63	1.30 (0.40)	4.77 (1.36)	136.59 (40.30)
70	1.31 (0.37)	4.82 (1.26)	138.70 (37.25)
77	1.33 (0.34)	4.88 (1.18)	140.73 (34.49)
84	1.34 (0.35)	4.94 (1.21)	143.04 (34.07)
365	1.49 (0.46)	5.51 (1.64)	155.29 (38.64)

Figures are forecast horizon (days), Mean Absolute Scaled Error (standard deviation), symmetric Mean Absolute Percentage Error (standard deviation) and Root Mean Squared Error (standard deviation) by forecast horizon ($n = 27$ folds)

### Model selection

The stage one MASE, 80 and 95% prediction interval coverage cross-validation results are summarised in supplementary Tables S1, S2, and S3 in the supplementary online material respectively. In the first stage of model selection, only Prophet (model 7) and the Prophet-Regression with ARIMA errors ensemble (model 13) had a MASE lower than 1.0 up to 84 day horizon ($MASE_{84}$ Prophet = 0.97 (0.12); ensemble = 0.99 (0.13)). In the ensemble the ARIMA model selected was a (1, 1, 3)(1, 0, 1, 7). The two models out performed seasonal naive at all horizons. All models had a MASE of greater than 1.0 at 365 days.

**Fig. 3:**
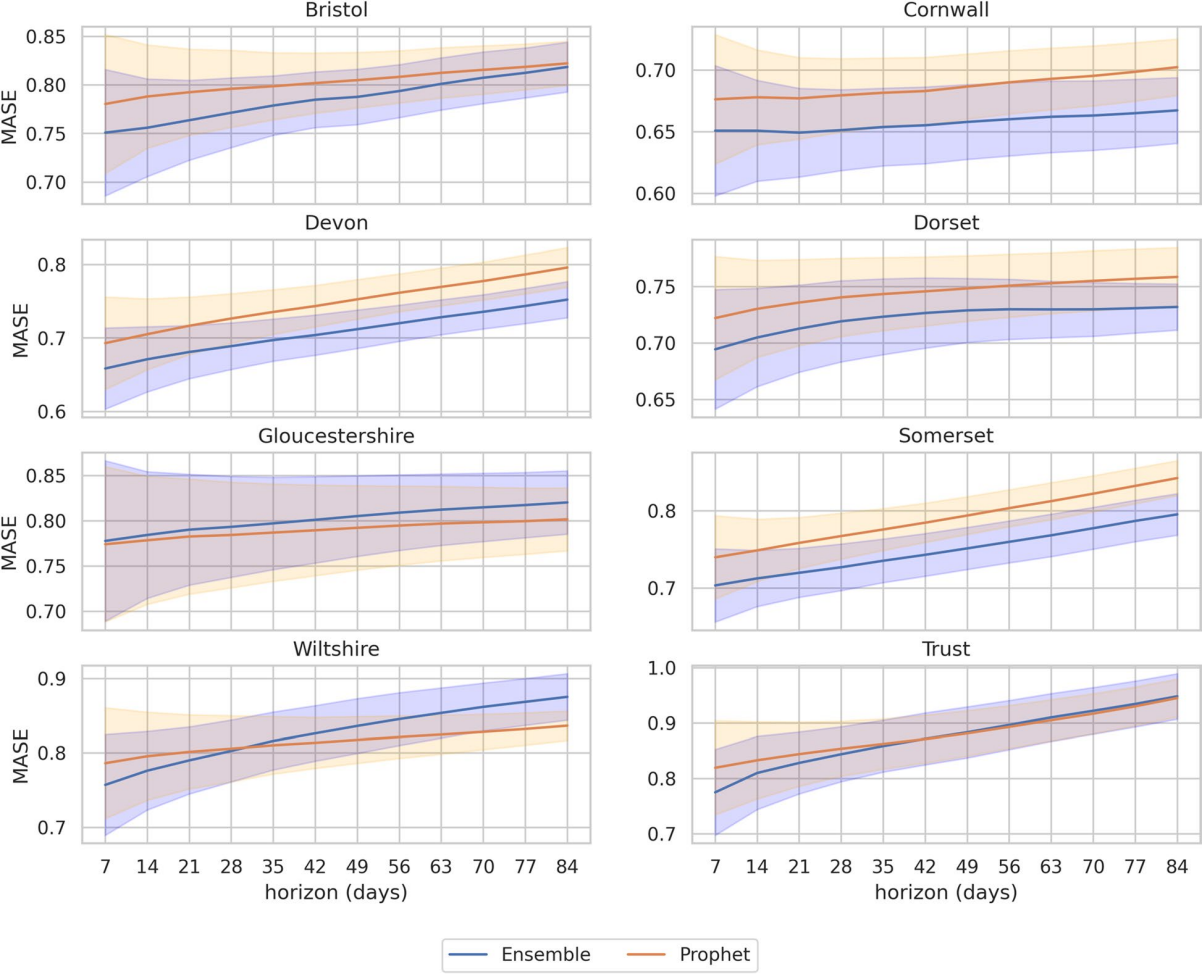
Cross-validation variation in MASE across regions by horizon. Shaded area is 95% prediction intervals for the mean point forecast error of the Prophet and Ensemble models

**Fig. 4:**
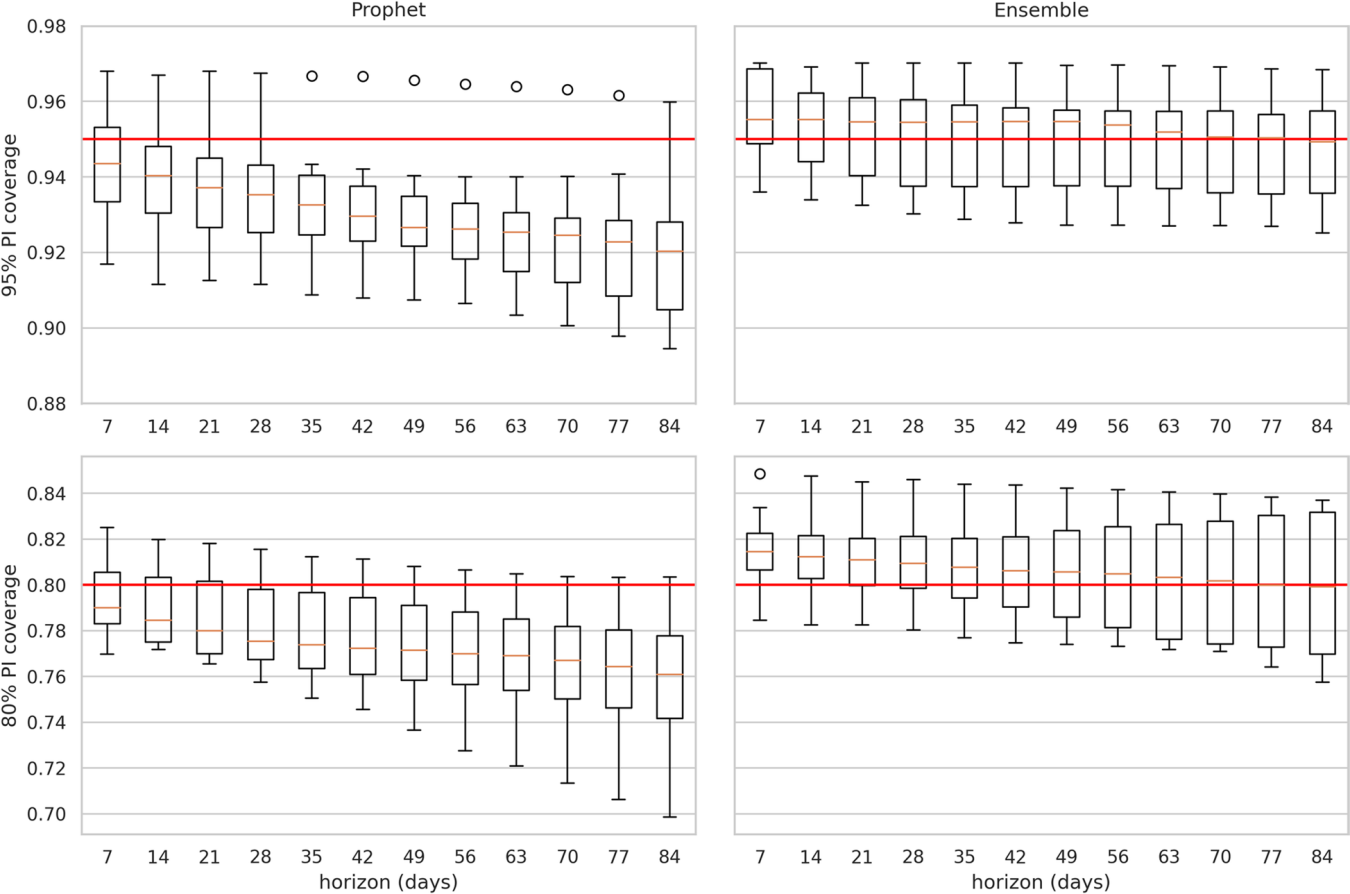
Cross-validation variation in Prediction Interval Coverage across regions by horizon. The box plots illustrate the distribution of coverage across all six regions. Red horizontal lines represent desired coverage. Top and bottom rows represents 95% and 80% prediction intervals, respectively. Left and right columns represent Prophet and the Ensemble, respectively. It is desirable to achieve coverage, but not exceed substantially

In the second stage of model selection, we compared Prophet and the ensemble at the sub region level. Figure 3 illustrates the change in MASE by forecast horizon and sub-region. Figure 4 illustrates the distribution of prediction interval coverage by horizon and desired coverage. The MASE for Prophet and the ensemble models is similar across all sub-regions. The median prediction interval coverage for the ensemble is more consistent than Prophet. The upper quartile of Prophet’s prediction interval coverage fails to achieve desired coverage from a horizon of 21 days. We chose the ensemble as our forecast benchmark model.

### Benchmark accuracy in the simulated forecast setting

Across all sub-regions and a horizon of 7 to 84 days the ensemble scored a benchmark MASE of 0.68 (95% CI 0.67 - 0.69) with 90% of the mean point forecast errors between 0.49 and 0.91 (an equivalent average error measured by sMAPE is 4.9%; 95% CI 4.7 - 5.1). Mean coverage for 80 and 95% prediction intervals was 0.847 (95% CI 0.843 - 0.851) and 0.965 (95% CI 0.949 - 0.977), respectively. Table 4 reports MASE and coverage for each forecast horizon. Table 5 provides detailed results of the average coverage for the 60th - 95th prediction intervals for each region.

**Table 4:** Benchmark results by forecast horizon

Horizon (days)	MASE	Coverage 80%	Coverage 95%
7	0.66 (0.64 - 0.69)	0.846 (0.829 - 0.864)	0.961 (0.952 - 0.970)
14	0.67 (0.65 - 0.69)	0.847 (0.835 - 0.860)	0.964 (0.958 - 0.970)
21	0.67 (0.65 - 0.68)	0.850 (0.839 - 0.860)	0.964 (0.959 - 0.970)
28	0.67 (0.66 - 0.69)	0.849 (0.840 - 0.858)	0.965 (0.961 - 0.969)
35	0.67 (0.66 - 0.69)	0.849 (0.841 - 0.858)	0.965 (0.961 - 0.969)
42	0.68 (0.66 - 0.69)	0.847 (0.839 - 0.855)	0.965 (0.962 - 0.969)
49	0.68 (0.67 - 0.69)	0.847 (0.839 - 0.855)	0.965 (0.962 - 0.969)
56	0.68 (0.67 - 0.70)	0.847 (0.839 - 0.854)	0.966 (0.963 - 0.969)
63	0.69 (0.68 - 0.70)	0.846 (0.839 - 0.854)	0.966 (0.964 - 0.969)
70	0.69 (0.68 - 0.70)	0.845 (0.838 - 0.853)	0.967 (0.964 - 0.969)
77	0.70 (0.68 - 0.71)	0.844 (0.837 - 0.852)	0.967 (0.964 - 0.969)
84	0.70 (0.69 - 0.71)	0.843 (0.835 - 0.851)	0.966 (0.963 - 0.969)

Mean (95% CI) MASE and 80, 95% prediction interval coverage. Figures pool all simulated forecasts from all seven sub-regions ($n = 287$; 41 folds per sub-region)

### External validation

In the 13 external validation sets the median number of responses ranged from 234 (IQR 222 - 246) to 752 (IQR 713 - 788). See supplementary Table S6 for additional summary measures. There were no missing data in the external validation set. Overall the ensemble produced a MASE of 0.73 (95% CI 0.72 - 0.74) with 90% of validation folds achieving a MASE between 0.64 and 0.83. The ensembles’ 80% and 95% prediction intervals provided 0.833 (95% CI 0.828-0.838) and 0.965 95% CI (0.963-0.967) coverage, respectively. Table 6 reports MASE and coverage by region.

## Discussion

Our chosen benchmark method, based on performance, is an ensemble (a simple average) of Facebook’s Prophet and Regression with ARIMA errors. Both methods are flexible enough to add in special calendar events such as national holidays. In our model we chose to include New Year’s day as this clearly stood out in the time series. In our regression model, we model the error process using the same ARIMA model - (1, 1, 3)(1, 0, 1, 7) - for each sub region. Other EMS providers in different regions can adopt this structure, but may wish to experiment with alternative ARIMA error processes for fine tuning.

**Table 5:** Prediction interval coverage by sub-region

Region	60%	70%	80%	90%	95%
BNSSG	0.639 (0.634 - 0.644)	0.746 (0.739 - 0.753)	0.844 (0.839 - 0.850)	0.922 (0.918 - 0.926)	0.959 (0.954 - 0.963)
Cornwall	0.637 (0.634 - 0.639)	0.762 (0.760 - 0.764)	0.856 (0.855 - 0.857)	0.921 (0.921 - 0.922)	0.974 (0.974 - 0.975)
Devon	0.691 (0.684 - 0.699)	0.793 (0.789 - 0.797)	0.857 (0.856 - 0.859)	0.939 (0.935 - 0.942)	0.974 (0.972 - 0.977)
Dorset	0.712 (0.708 - 0.715)	0.803 (0.800 - 0.805)	0.873 (0.871 - 0.876)	0.940 (0.938 - 0.941)	0.975 (0.974 - 0.976)
Glouc	0.626 (0.621 - 0.632)	0.730 (0.728 - 0.733)	0.835 (0.833 - 0.837)	0.936 (0.934 - 0.937)	0.963 (0.962 - 0.964)
Somerset	0.629 (0.625 - 0.633)	0.723 (0.718 - 0.728)	0.809 (0.805 - 0.813)	0.901 (0.899 - 0.903)	0.950 (0.948 - 0.951)
Wiltshire	0.665 (0.662 - 0.668)	0.762 (0.760 - 0.765)	0.853 (0.847 - 0.859)	0.928 (0.925 - 0.932)	0.961 (0.960 - 0.963)

Mean (95% CI) prediction interval coverage. Figures relate to 41 folds of 7-84 days in the test set

**Table 6:** External validation: Point forecast and coverage performance by region

Trust	Region	MASE	Coverage 80	Coverage 95
London	North Central	0.76 (0.11)	0.84 (0.06)	0.94 (0.04)
	North East	0.74 (0.08)	0.87 (0.06)	0.97 (0.03)
	North West	0.75 (0.11)	0.86 (0.07)	0.97 (0.03)
	South East	0.70 (0.10)	0.85 (0.06)	0.98 (0.03)
	South West	0.68 (0.10)	0.86 (0.06)	0.97 (0.04)
Wales	Central and West	0.69 (0.12)	0.82 (0.09)	0.96 (0.04)
	North	0.73 (0.14)	0.79 (0.10)	0.97 (0.04)
	South East	0.64 (0.10)	0.87 (0.06)	0.98 (0.04)
Yorkshire	ABL	0.67 (0.11)	0.86 (0.05)	0.98 (0.02)
	CKW	0.74 (0.09)	0.82 (0.07)	0.96 (0.03)
	Humb and ER	0.77 (0.11)	0.79 (0.08)	0.94 (0.04)
	North Yorks	0.83 (0.15)	0.77 (0.09)	0.94 (0.06)
	South	0.76 (0.11)	0.81 (0.08)	0.96 (0.04)

Mean (SD). Figures calculated from 41 folds of 7-84 days in test sets

Our cross-validation demonstrated that performance of the ensemble was superior to either method on its own, the other candidate models and a naive benchmark. However, we note that Prophet is also a reasonable choice for ambulance trusts new to forecasting (albeit they should recognise the shortcomings in terms of coverage). We emphasise the critical importance of a naive benchmark such as seasonal naive in cross-validation to confirm that more complex models add value. We found that over our forecast horizon seasonal naive outperformed several state-of-the-art forecasting techniques. We encourage forecasters in the ambulance service to use both point forecasts and prediction intervals. A singular focus on a point forecast is unwise; it is not possible to predict the future exactly and so forecasters should take account of the range of likely values. We found that the most accurate single method for point forecasts did not produce satisfactory coverage. The software we have developed to support forecasting in ambulance services reports 95% prediction intervals by default.

Turning to benchmark performance, our simulated forecast achieved a MASE of 0.68. We found this performance declined slightly over the forecast horizon, and 90% of forecasts fell into range of 0.49 and 0.91. The latter is a reasonable approximation for practitioners to use as a rule of thumb for benchmarking. This is evidenced by our external validation of the model. Using data from London, Yorkshire and Wales we found forecast performance within the benchmarks expected range (where 90% of MASE scores fell between 0.64 and 0.83). Researchers should make use of our detailed breakdown of MASE to enable simple robust comparison across regions and studies. We emphasise that in ‘real terms’ for an individual ambulance service our forecasts demonstrate a useful accuracy up to 84 days. For instance, the RMSE for a 84 day horizon in Cornwall (average calls per day = 243) and Dorset (average = 323) was between 16 and 18 calls. Our results are also complementary to Martin et al’s [12] daily EMS time series predictions that achieved a mean absolute percentage error (MAPE) of 5.9%. Our sMAPE results were inline with these findings with an overall mean of 4.9% over the 84 day forecast horizon.

Our ensemble method achieved desired coverage, but we acknowledge that it is conservative at the 80% level. We found that coverage varied by sub-region and advise practitioners to investigate their own time series. A benchmark coverage for 84% and 96% was achieved for the 80 and 95% prediction intervals, respectively. We provide a more detailed breakdown of coverage in Table 4 for future scientific forecasting studies. Our detailed results are also relevant to studies that aim to building forecasting into decision support systems [30]. Further work may wish to explore other methods for measuring prediction interval uncertainty, such as the Winkler score [31], relative to our measure of empirical coverage.

### Practical implications

The ensemble model has been implemented by the ambulance service covering the South West of England. The EMS data science team have also adapted the model to predict the daily count of calls received by their clinical call centre in order to support staffing decisions.

### Strengths of the study

There are several strengths to our study relative to existing studies. First, our model selection focused on well-known forecasting methods including a recognised and relevant naive benchmark. We excluded novel methods development from our study as we wished our method to be widely available and easily implemented in a health service - particularly the UK’s NHS where the study setting is based. Indeed the methods in our ensemble are recognised by NHS Improvement for forecasting [32]. Our method is implemented and available in Python and can easily be implemented in R. Second, we based our benchmark accuracy on the performance of the ensemble on seven time series from regions in South West of England. The results were replicated in 13 regions from London, Wales and Yorkshire. This evidence provides a robust estimate of what can be expected in practice and a strong comparator for future studies attempting to improve on our ensemble. Third, we include prediction interval coverage as a primary outcome in addition to point forecast accuracy. Both our external validation and assessment of prediction coverage is a substantial step up from existing high quality studies [5, 8].

### Limitations

Our study also has several limitations. Our geographic regions are limited in number and within England and Wales only. Other geographic regions of the UK and other nations may have differing seasonal patterns. We argue that two elements of our study mitigate this limitation to some extent. First our approach makes use of automatic modelling procedures that enable deployment at scale and manage some issues with differences in seasonality. Second, our primary objective is clear benchmark performance. Other regions can easily compare their outcomes to our own. Our data run until January 2020 and takes place prior to the COVID-19 pandemic. In this time period emergency services around the world will have seen dramatic shifts in their demand and a potential increase in the importance of weather. Assuming demand returns to similar patterns post-pandemic our ensemble should be viable with some minor modification. For instance, both Prophet and Regression with ARIMA errors can be modified to include binary ‘intervention‘ variables that can represent different phases of the pandemic. Prophet’s flexible modelling of trend also allows for manual correction at critical time points (e.g. the start and end of lockdowns). A further limitation is that our methods do not make use of other features that may aid short term prediction such as weather forecasts.

## Conclusions

The primary contribution of our study is the benchmark performance for predicting the number of calls that result in the dispatch of one or more ambulances. We provide externally validated estimates up to 84 days in advance. Future studies and novel methodologies should now aim to exceed these benchmarks. A potential future direction is to compare the neural network architectures successfully applied in related studies [6, 11, 12] to our benchmark. We encourage future research that aims to predict the daily number EMS calls that result in the dispatch of one or more ambulances to consider our results as a reliable benchmark for their methods.

## Supplementary Information

**Additional file 1.** Supplementary Results. Additional large tables of results for model selection and data description.

## Data Availability

The code and data used within this study can be freely and openly access via the Zenodo repository [25] https://doi.org/10.5281/zenodo.4850149.
