# Supplementary material for: Forecasting the daily demand for emergency medical ambulances in England and Wales: a benchmark model and external validation
Source: BMC Med Inform Decis Mak. 2023 Jul 11;23:117. doi: 10.1186/s12911-023-02218-z (PMC10334567; doi:10.1186/s12911-023-02218-z)
Supplement: Supplementary file 1 — Additional file 1. Supplementary Results. Additional large tables of results for model selection and data description. [file 12911_2023_2218_MOESM1_ESM.docx]

Supplementary Results: Model Selection

Tables S1-S6 report the cross validation results. For reference see Table 1 in the main paper that identifies forecasting methods by number. The numbering corresponds to the header in the cross validation result tables. Models 9 and 13 were selected from stage one.

1

2

| h | naive | 1 | 2 | 3 | 4 | 5 | 6 | 7 | 8 | 9 | 10 | 11 | 12 | 13 | 14 |
| --- | --- | --- | --- | --- | --- | --- | --- | --- | --- | --- | --- | --- | --- | --- | --- |
| 7 | 0.94 (0.35) | 0.78 (0.37) | 0.82 (0.39) | 0.86 (0.32) | 0.76 (0.28) | 1.21 (0.67) | 0.88 (0.34) | 0.88 (0.46) | 1.01 (0.46) | 0.81 (0.42) | 0.85 (0.59) | 1.12 (0.45) | 0.77 (0.33) | 0.78 (0.34) | 0.77 (0.35) |
| 14 | 1.06 (0.39) | 0.88 (0.43) | 0.92 (0.40) | 0.96 (0.31) | 0.85 (0.29) | 1.62 (0.90) | 0.97 (0.31) | 0.97 (0.44) | 1.07 (0.42) | 0.82 (0.36) | 0.95 (0.63) | 1.19 (0.44) | 0.87 (0.36) | 0.83 (0.32) | 0.84 (0.35) |
| 21 | 1.12 (0.41) | 0.92 (0.42) | 0.97 (0.37) | 1.02 (0.27) | 0.91 (0.29) | 1.88 (1.02) | 1.03 (0.26) | 1.03 (0.38) | 1.13 (0.40) | 0.83 (0.29) | 1.01 (0.68) | 1.22 (0.41) | 0.91 (0.35) | 0.85 (0.26) | 0.86 (0.30) |
| 28 | 1.14 (0.40) | 0.95 (0.39) | 1.01 (0.36) | 1.07 (0.27) | 0.96 (0.28) | 2.08 (1.09) | 1.09 (0.25) | 1.07 (0.35) | 1.16 (0.39) | 0.83 (0.23) | 1.05 (0.68) | 1.24 (0.37) | 0.94 (0.33) | 0.86 (0.20) | 0.88 (0.25) |
| 35 | 1.18 (0.40) | 1.00 (0.40) | 1.05 (0.37) | 1.12 (0.29) | 1.00 (0.29) | 2.27 (1.17) | 1.14 (0.27) | 1.12 (0.41) | 1.19 (0.40) | 0.84 (0.20) | 1.10 (0.69) | 1.28 (0.37) | 0.98 (0.34) | 0.88 (0.17) | 0.91 (0.23) |
| 42 | 1.23 (0.40) | 1.04 (0.40) | 1.08 (0.37) | 1.15 (0.31) | 1.05 (0.31) | 2.44 (1.23) | 1.17 (0.27) | 1.17 (0.43) | 1.21 (0.40) | 0.84 (0.18) | 1.15 (0.69) | 1.31 (0.38) | 1.02 (0.34) | 0.90 (0.17) | 0.93 (0.23) |
| 49 | 1.25 (0.39) | 1.08 (0.40) | 1.11 (0.36) | 1.19 (0.32) | 1.08 (0.31) | 2.60 (1.22) | 1.20 (0.27) | 1.20 (0.43) | 1.23 (0.40) | 0.85 (0.16) | 1.18 (0.69) | 1.34 (0.36) | 1.05 (0.35) | 0.91 (0.16) | 0.95 (0.22) |
| 56 | 1.27 (0.39) | 1.10 (0.38) | 1.13 (0.34) | 1.22 (0.32) | 1.11 (0.30) | 2.74 (1.16) | 1.23 (0.27) | 1.24 (0.43) | 1.25 (0.40) | 0.86 (0.15) | 1.19 (0.68) | 1.36 (0.34) | 1.07 (0.34) | 0.93 (0.14) | 0.97 (0.21) |
| 63 | 1.30 (0.40) | 1.13 (0.39) | 1.15 (0.33) | 1.25 (0.33) | 1.14 (0.31) | 2.87 (1.10) | 1.25 (0.26) | 1.27 (0.43) | 1.27 (0.38) | 0.87 (0.13) | 1.22 (0.66) | 1.39 (0.34) | 1.10 (0.33) | 0.95 (0.13) | 0.99 (0.21) |
| 70 | 1.31 (0.37) | 1.15 (0.36) | 1.17 (0.31) | 1.28 (0.33) | 1.17 (0.31) | 2.97 (1.01) | 1.27 (0.26) | 1.30 (0.44) | 1.29 (0.36) | 0.87 (0.12) | 1.23 (0.65) | 1.42 (0.32) | 1.12 (0.30) | 0.96 (0.12) | 1.01 (0.19) |
| 77 | 1.33 (0.34) | 1.17 (0.33) | 1.18 (0.29) | 1.31 (0.33) | 1.19 (0.31) | 3.07 (0.90) | 1.29 (0.26) | 1.33 (0.44) | 1.31 (0.34) | 0.88 (0.12) | 1.24 (0.62) | 1.44 (0.29) | 1.14 (0.28) | 0.97 (0.11) | 1.02 (0.17) |
| 84 | 1.34 (0.35) | 1.19 (0.33) | 1.20 (0.30) | 1.34 (0.33) | 1.21 (0.33) | 3.18 (0.84) | 1.32 (0.26) | 1.37 (0.44) | 1.33 (0.33) | 0.90 (0.11) | 1.25 (0.60) | 1.47 (0.29) | 1.16 (0.29) | 0.99 (0.13) | 1.04 (0.18) |
| 365 | 1.49 (0.46) | 1.39 (0.46) | 1.34 (0.41) | 1.33 (0.06) | 1.52 (0.59) | 3.05 (0.95) | 1.33 (0.07) | 1.60 (0.41) | 1.44 (0.12) | 1.15 (0.20) | 1.37 (0.49) | 1.65 (0.47) | 1.39 (0.47) | 1.26 (0.33) | 1.29 (0.37) |

Table S1: Results for stage 1 model selection. Mean Absolute Scaled Error (MASE) of Point Forecasts (Stdev). The numeric numbering of columns references the list of methods in Table 1.

|  | 1 | 2 | 3 | 4 | 5 | 6 | 7 | 8 | 9 | 10 | 11 | 12 | 13 | 14 |
| --- | --- | --- | --- | --- | --- | --- | --- | --- | --- | --- | --- | --- | --- | --- |
| 7 | 0.85 (0.19) | 0.81 (0.20) | 0.67 (0.21) | 0.80 (0.18) | 0.78 (0.29) | 0.68 (0.23) | 0.80 (0.23) | 0.70 (0.27) | 0.78 (0.20) | 0.84 (0.23) | 0.84 (0.14) | 0.83 (0.21) | 0.79 (0.21) | 0.82 (0.20) |
| 14 | 0.86 (0.18) | 0.78 (0.17) | 0.61 (0.18) | 0.79 (0.16) | 0.76 (0.28) | 0.61 (0.19) | 0.78 (0.20) | 0.67 (0.25) | 0.77 (0.17) | 0.85 (0.23) | 0.91 (0.10) | 0.82 (0.19) | 0.78 (0.18) | 0.81 (0.19) |
| 21 | 0.87 (0.16) | 0.77 (0.15) | 0.57 (0.15) | 0.77 (0.15) | 0.74 (0.27) | 0.57 (0.16) | 0.78 (0.15) | 0.67 (0.24) | 0.76 (0.14) | 0.86 (0.22) | 0.94 (0.06) | 0.83 (0.17) | 0.78 (0.14) | 0.82 (0.16) |
| 28 | 0.88 (0.14) | 0.76 (0.14) | 0.54 (0.14) | 0.74 (0.14) | 0.71 (0.27) | 0.54 (0.14) | 0.76 (0.14) | 0.66 (0.25) | 0.76 (0.12) | 0.87 (0.21) | 0.95 (0.05) | 0.84 (0.14) | 0.78 (0.11) | 0.83 (0.13) |
| 35 | 0.89 (0.14) | 0.75 (0.15) | 0.52 (0.15) | 0.72 (0.14) | 0.69 (0.28) | 0.52 (0.15) | 0.75 (0.16) | 0.66 (0.26) | 0.77 (0.11) | 0.88 (0.21) | 0.95 (0.05) | 0.84 (0.14) | 0.77 (0.10) | 0.83 (0.13) |
| 42 | 0.90 (0.14) | 0.74 (0.15) | 0.50 (0.15) | 0.71 (0.14) | 0.66 (0.28) | 0.50 (0.16) | 0.73 (0.16) | 0.66 (0.26) | 0.76 (0.10) | 0.89 (0.20) | 0.96 (0.04) | 0.84 (0.14) | 0.77 (0.10) | 0.84 (0.12) |
| 49 | 0.90 (0.12) | 0.73 (0.15) | 0.49 (0.15) | 0.70 (0.14) | 0.64 (0.29) | 0.49 (0.15) | 0.72 (0.17) | 0.66 (0.25) | 0.76 (0.10) | 0.89 (0.20) | 0.97 (0.03) | 0.85 (0.13) | 0.76 (0.09) | 0.84 (0.11) |
| 56 | 0.91 (0.10) | 0.73 (0.14) | 0.47 (0.14) | 0.69 (0.14) | 0.63 (0.28) | 0.47 (0.15) | 0.71 (0.18) | 0.65 (0.24) | 0.76 (0.10) | 0.90 (0.19) | 0.97 (0.03) | 0.85 (0.12) | 0.76 (0.08) | 0.84 (0.10) |
| 63 | 0.91 (0.10) | 0.72 (0.14) | 0.45 (0.14) | 0.69 (0.15) | 0.62 (0.26) | 0.46 (0.14) | 0.70 (0.18) | 0.65 (0.24) | 0.76 (0.09) | 0.90 (0.18) | 0.97 (0.03) | 0.86 (0.12) | 0.75 (0.08) | 0.84 (0.09) |
| 70 | 0.92 (0.09) | 0.72 (0.13) | 0.44 (0.14) | 0.68 (0.15) | 0.61 (0.23) | 0.45 (0.14) | 0.69 (0.18) | 0.64 (0.23) | 0.76 (0.09) | 0.91 (0.18) | 0.97 (0.03) | 0.86 (0.11) | 0.75 (0.07) | 0.85 (0.09) |
| 77 | 0.92 (0.09) | 0.72 (0.12) | 0.43 (0.14) | 0.68 (0.15) | 0.60 (0.21) | 0.43 (0.13) | 0.69 (0.18) | 0.63 (0.22) | 0.75 (0.09) | 0.91 (0.17) | 0.97 (0.02) | 0.86 (0.10) | 0.74 (0.07) | 0.85 (0.08) |
| 84 | 0.93 (0.08) | 0.71 (0.12) | 0.41 (0.13) | 0.67 (0.16) | 0.58 (0.20) | 0.42 (0.12) | 0.67 (0.18) | 0.62 (0.21) | 0.75 (0.09) | 0.92 (0.16) | 0.98 (0.02) | 0.87 (0.10) | 0.73 (0.09) | 0.85 (0.08) |
| 365 | 0.97 (0.02) | 0.78 (0.11) | 0.43 (0.04) | 0.73 (0.16) | 0.79 (0.12) | 0.45 (0.04) | 0.63 (0.19) | 0.59 (0.11) | 0.67 (0.10) | 0.98 (0.03) | 0.99 (0.00) | 0.94 (0.06) | 0.72 (0.13) | 0.91 (0.07) |

Table S2: Cross Validation of 80% Prediction Interval Coverage (cv=27)

3

|  | 1 | 2 | 3 | 4 | 5 | 6 | 7 | 8 | 9 | 10 | 11 | 12 | 13 | 14 |
| --- | --- | --- | --- | --- | --- | --- | --- | --- | --- | --- | --- | --- | --- | --- |
| 7 | 0.97 (0.07) | 0.96 (0.09) | 0.90 (0.15) | 0.96 (0.08) | 0.93 (0.16) | 0.89 (0.16) | 0.94 (0.15) | 0.90 (0.19) | 0.92 (0.14) | 0.97 (0.06) | 0.96 (0.08) | 0.97 (0.07) | 0.94 (0.13) | 0.94 (0.13) |
| 14 | 0.96 (0.11) | 0.95 (0.11) | 0.86 (0.16) | 0.94 (0.11) | 0.91 (0.18) | 0.85 (0.17) | 0.94 (0.12) | 0.91 (0.16) | 0.91 (0.11) | 0.97 (0.08) | 0.97 (0.05) | 0.95 (0.11) | 0.93 (0.11) | 0.92 (0.14) |
| 21 | 0.96 (0.10) | 0.95 (0.10) | 0.83 (0.15) | 0.93 (0.11) | 0.90 (0.19) | 0.82 (0.15) | 0.94 (0.10) | 0.91 (0.15) | 0.91 (0.09) | 0.97 (0.06) | 0.98 (0.04) | 0.95 (0.11) | 0.92 (0.10) | 0.93 (0.11) |
| 28 | 0.97 (0.08) | 0.94 (0.09) | 0.80 (0.14) | 0.93 (0.10) | 0.89 (0.20) | 0.79 (0.13) | 0.94 (0.09) | 0.91 (0.14) | 0.91 (0.08) | 0.98 (0.05) | 0.99 (0.03) | 0.95 (0.09) | 0.92 (0.08) | 0.94 (0.09) |
| 35 | 0.97 (0.07) | 0.94 (0.09) | 0.78 (0.14) | 0.92 (0.10) | 0.89 (0.19) | 0.77 (0.14) | 0.93 (0.09) | 0.91 (0.14) | 0.91 (0.07) | 0.98 (0.04) | 0.99 (0.02) | 0.95 (0.08) | 0.92 (0.07) | 0.94 (0.08) |
| 42 | 0.98 (0.05) | 0.94 (0.08) | 0.75 (0.15) | 0.91 (0.10) | 0.88 (0.19) | 0.75 (0.14) | 0.92 (0.09) | 0.91 (0.14) | 0.91 (0.07) | 0.98 (0.03) | 0.99 (0.02) | 0.96 (0.07) | 0.92 (0.06) | 0.94 (0.07) |
| 49 | 0.98 (0.05) | 0.93 (0.08) | 0.73 (0.16) | 0.91 (0.09) | 0.87 (0.19) | 0.74 (0.14) | 0.91 (0.09) | 0.91 (0.14) | 0.91 (0.06) | 0.98 (0.03) | 0.99 (0.02) | 0.96 (0.06) | 0.92 (0.06) | 0.95 (0.06) |
| 56 | 0.98 (0.04) | 0.93 (0.08) | 0.71 (0.16) | 0.90 (0.08) | 0.88 (0.16) | 0.72 (0.13) | 0.90 (0.10) | 0.91 (0.14) | 0.91 (0.06) | 0.98 (0.02) | 0.99 (0.01) | 0.96 (0.06) | 0.92 (0.05) | 0.95 (0.06) |
| 63 | 0.98 (0.04) | 0.93 (0.07) | 0.70 (0.16) | 0.89 (0.09) | 0.87 (0.16) | 0.71 (0.13) | 0.89 (0.11) | 0.91 (0.12) | 0.90 (0.06) | 0.99 (0.02) | 0.99 (0.01) | 0.96 (0.05) | 0.92 (0.04) | 0.95 (0.05) |
| 70 | 0.98 (0.03) | 0.92 (0.06) | 0.68 (0.16) | 0.89 (0.09) | 0.87 (0.15) | 0.70 (0.13) | 0.88 (0.12) | 0.91 (0.11) | 0.90 (0.05) | 0.99 (0.02) | 0.99 (0.01) | 0.96 (0.05) | 0.92 (0.04) | 0.95 (0.05) |
| 77 | 0.98 (0.03) | 0.92 (0.06) | 0.67 (0.16) | 0.88 (0.09) | 0.87 (0.13) | 0.69 (0.13) | 0.88 (0.13) | 0.91 (0.10) | 0.90 (0.05) | 0.99 (0.02) | 0.99 (0.01) | 0.96 (0.04) | 0.92 (0.03) | 0.95 (0.04) |
| 84 | 0.98 (0.03) | 0.92 (0.06) | 0.66 (0.16) | 0.88 (0.10) | 0.87 (0.13) | 0.68 (0.12) | 0.87 (0.14) | 0.91 (0.09) | 0.90 (0.05) | 0.99 (0.02) | 0.99 (0.01) | 0.96 (0.04) | 0.91 (0.03) | 0.95 (0.04) |
| 365 | 0.99 (0.01) | 0.94 (0.04) | 0.68 (0.03) | 0.91 (0.06) | 0.93 (0.06) | 0.69 (0.03) | 0.84 (0.11) | 0.87 (0.05) | 0.88 (0.05) | 1.00 (0.00) | 1.00 (0.00) | 0.98 (0.01) | 0.92 (0.05) | 0.98 (0.01) |

Table S3:Cross Validation of 95% Prediction Interval Coverage (cv=27)

|  | naive | 1 | 2 | 3 | 4 | 5 | 6 | 7 | 8 | 9 | 10 | 11 | 12 | 13 | 14 |
| --- | --- | --- | --- | --- | --- | --- | --- | --- | --- | --- | --- | --- | --- | --- | --- |
| 7 | 3.48 (1.19) | 2.87 (1.24) | 3.04 (1.26) | 3.21 (1.12) | 2.82 (0.96) | 4.57 (2.49) | 3.26 (1.20) | 3.23 (1.50) | 3.73 (1.57) | 2.99 (1.45) | 3.12 (2.04) | 4.14 (1.49) | 2.84 (1.09) | 2.89 (1.15) | 2.85 (1.16) |
| 14 | 3.94 (1.32) | 3.23 (1.44) | 3.39 (1.28) | 3.56 (1.05) | 3.16 (0.96) | 6.10 (3.13) | 3.63 (1.06) | 3.59 (1.45) | 3.96 (1.44) | 3.05 (1.25) | 3.48 (2.16) | 4.39 (1.43) | 3.19 (1.19) | 3.07 (1.08) | 3.09 (1.17) |
| 21 | 4.12 (1.38) | 3.39 (1.40) | 3.58 (1.19) | 3.80 (0.88) | 3.38 (0.95) | 7.04 (3.46) | 3.85 (0.89) | 3.79 (1.22) | 4.16 (1.38) | 3.08 (1.01) | 3.71 (2.36) | 4.48 (1.33) | 3.35 (1.15) | 3.14 (0.85) | 3.18 (1.00) |
| 28 | 4.19 (1.33) | 3.50 (1.30) | 3.73 (1.14) | 4.00 (0.86) | 3.53 (0.88) | 7.80 (3.65) | 4.07 (0.83) | 3.96 (1.14) | 4.29 (1.38) | 3.09 (0.82) | 3.85 (2.37) | 4.57 (1.16) | 3.46 (1.06) | 3.18 (0.63) | 3.24 (0.80) |
| 35 | 4.35 (1.34) | 3.66 (1.31) | 3.87 (1.18) | 4.16 (0.96) | 3.70 (0.94) | 8.51 (3.99) | 4.24 (0.91) | 4.15 (1.39) | 4.40 (1.40) | 3.10 (0.73) | 4.02 (2.41) | 4.71 (1.21) | 3.61 (1.10) | 3.24 (0.55) | 3.33 (0.75) |
| 42 | 4.51 (1.33) | 3.82 (1.32) | 3.98 (1.19) | 4.30 (1.03) | 3.85 (0.99) | 9.18 (4.21) | 4.37 (0.94) | 4.31 (1.48) | 4.47 (1.44) | 3.12 (0.68) | 4.19 (2.44) | 4.83 (1.24) | 3.75 (1.13) | 3.31 (0.52) | 3.42 (0.74) |
| 49 | 4.61 (1.32) | 3.94 (1.31) | 4.07 (1.17) | 4.43 (1.07) | 3.98 (1.02) | 9.78 (4.20) | 4.47 (0.94) | 4.44 (1.49) | 4.52 (1.44) | 3.14 (0.63) | 4.29 (2.43) | 4.91 (1.18) | 3.85 (1.14) | 3.36 (0.49) | 3.49 (0.72) |
| 56 | 4.67 (1.33) | 4.03 (1.28) | 4.14 (1.12) | 4.57 (1.11) | 4.09 (0.98) | 10.32 (3.99) | 4.57 (0.91) | 4.58 (1.48) | 4.59 (1.41) | 3.16 (0.58) | 4.36 (2.39) | 5.00 (1.14) | 3.94 (1.11) | 3.42 (0.44) | 3.57 (0.68) |
| 63 | 4.77 (1.36) | 4.14 (1.30) | 4.22 (1.09) | 4.67 (1.12) | 4.20 (1.05) | 10.83 (3.75) | 4.65 (0.90) | 4.70 (1.52) | 4.66 (1.35) | 3.19 (0.54) | 4.44 (2.33) | 5.12 (1.14) | 4.04 (1.11) | 3.48 (0.42) | 3.65 (0.68) |
| 70 | 4.82 (1.26) | 4.21 (1.20) | 4.27 (1.02) | 4.78 (1.15) | 4.29 (1.06) | 11.25 (3.43) | 4.72 (0.90) | 4.81 (1.54) | 4.73 (1.27) | 3.22 (0.53) | 4.49 (2.29) | 5.21 (1.08) | 4.11 (1.01) | 3.52 (0.39) | 3.70 (0.61) |
| 77 | 4.88 (1.18) | 4.27 (1.11) | 4.32 (0.98) | 4.89 (1.17) | 4.37 (1.06) | 11.65 (3.06) | 4.82 (0.90) | 4.92 (1.56) | 4.80 (1.19) | 3.25 (0.52) | 4.52 (2.21) | 5.29 (1.01) | 4.17 (0.94) | 3.56 (0.39) | 3.75 (0.56) |
| 84 | 4.94 (1.21) | 4.35 (1.13) | 4.40 (1.00) | 4.99 (1.17) | 4.45 (1.15) | 12.07 (2.89) | 4.90 (0.86) | 5.04 (1.56) | 4.89 (1.15) | 3.29 (0.51) | 4.56 (2.13) | 5.39 (1.05) | 4.25 (0.97) | 3.63 (0.46) | 3.82 (0.62) |
| 365 | 5.51 (1.64) | 5.12 (1.67) | 4.94 (1.45) | 5.00 (0.22) | 5.58 (2.04) | 11.50 (3.44) | 4.97 (0.25) | 5.99 (1.60) | 5.38 (0.48) | 4.23 (0.71) | 5.04 (1.77) | 6.12 (1.75) | 5.11 (1.65) | 4.62 (1.18) | 4.75 (1.32) |

Table S4: symmetric MAPE point forecast results (cv=27)

4

|  | naive | 1 | 2 | 3 | 4 | 5 | 6 | 7 | 8 | 9 | 10 | 11 | 12 | 13 | 14 |
| --- | --- | --- | --- | --- | --- | --- | --- | --- | --- | --- | --- | --- | --- | --- | --- |
| 7 | 97 (37) | 77 (37) | 81 (39) | 83 (28) | 74 (25) | 114 (57) | 85 (29) | 87 (48) | 100 (46) | 81 (46) | 83 (52) | 116 (42) | 75 (28) | 76 (32) | 76 (32) |
| 14 | 110 (42) | 88 (43) | 93 (42) | 94 (29) | 85 (27) | 157 (84) | 96 (29) | 99 (46) | 108 (43) | 86 (41) | 94 (57) | 125 (42) | 86 (33) | 83 (31) | 83 (34) |
| 21 | 115 (43) | 94 (42) | 100 (41) | 102 (27) | 92 (27) | 183 (93) | 103 (26) | 106 (42) | 114 (42) | 89 (36) | 102 (61) | 130 (39) | 91 (33) | 87 (28) | 88 (32) |
| 28 | 118 (43) | 98 (40) | 105 (40) | 108 (27) | 97 (26) | 203 (97) | 109 (25) | 112 (39) | 119 (41) | 91 (32) | 107 (61) | 134 (37) | 95 (31) | 90 (24) | 91 (28) |
| 35 | 123 (42) | 104 (40) | 110 (41) | 112 (28) | 101 (27) | 220 (103) | 114 (25) | 117 (43) | 122 (41) | 92 (29) | 112 (60) | 138 (37) | 100 (32) | 92 (22) | 94 (27) |
| 42 | 128 (40) | 108 (39) | 113 (40) | 115 (29) | 106 (27) | 237 (107) | 117 (25) | 122 (45) | 125 (41) | 94 (26) | 117 (60) | 142 (38) | 104 (32) | 94 (21) | 98 (26) |
| 49 | 131 (40) | 112 (39) | 117 (40) | 118 (29) | 110 (28) | 251 (106) | 120 (24) | 126 (44) | 127 (41) | 95 (24) | 121 (60) | 146 (36) | 107 (33) | 96 (19) | 100 (25) |
| 56 | 134 (40) | 115 (38) | 119 (39) | 121 (29) | 113 (27) | 264 (101) | 122 (23) | 130 (44) | 130 (41) | 96 (21) | 123 (59) | 149 (35) | 110 (32) | 98 (18) | 102 (24) |
| 63 | 137 (40) | 118 (38) | 122 (37) | 124 (29) | 116 (28) | 278 (94) | 124 (23) | 134 (44) | 132 (39) | 98 (18) | 126 (58) | 152 (34) | 114 (32) | 100 (16) | 105 (23) |
| 70 | 139 (37) | 121 (36) | 125 (35) | 126 (29) | 119 (29) | 289 (84) | 126 (23) | 137 (44) | 135 (38) | 99 (15) | 128 (56) | 155 (32) | 116 (30) | 102 (14) | 107 (20) |
| 77 | 141 (34) | 123 (33) | 127 (34) | 129 (29) | 121 (28) | 300 (71) | 128 (23) | 140 (44) | 137 (37) | 100 (13) | 130 (53) | 158 (28) | 118 (27) | 103 (12) | 109 (18) |
| 84 | 143 (34) | 126 (32) | 129 (34) | 132 (29) | 124 (31) | 311 (64) | 130 (23) | 144 (44) | 140 (36) | 101 (11) | 131 (51) | 160 (27) | 121 (28) | 106 (13) | 111 (18) |
| 365 | 155 (39) | 144 (37) | 142 (33) | 134 (4) | 154 (48) | 314 (79) | 133 (5) | 164 (33) | 152 (12) | 122 (17) | 142 (39) | 173 (40) | 143 (38) | 129 (28) | 133 (30) |

Table S5: RMSE point forecast results (cv=27)

5

|  | Trust | Sub-region | n | mean | std | min | 25% | 50% | 75% | max |
| --- | --- | --- | --- | --- | --- | --- | --- | --- | --- | --- |
| 0 |  | BNSSG | 1828.0 | 475.0 | 40.0 | 374.0 | 447.0 | 474.0 | 499.0 | 662.0 |
| 1 |  | Cornwall | 1828.0 | 242.0 | 21.0 | 172.0 | 228.0 | 242.0 | 256.0 | 341.0 |
| 2 |  | Devon | 1828.0 | 514.0 | 37.0 | 407.0 | 490.0 | 512.0 | 538.0 | 774.0 |
| 3 | South West | Dorset | 1828.0 | 324.0 | 26.0 | 248.0 | 306.0 | 322.0 | 338.0 | 571.0 |
| 4 |  | Gloucestershire | 1828.0 | 139.0 | 16.0 | 94.0 | 128.0 | 138.0 | 149.0 | 231.0 |
| 5 |  | Somerset | 1828.0 | 191.0 | 17.0 | 146.0 | 179.0 | 191.0 | 203.0 | 255.0 |
| 6 |  | Wiltshire | 1828.0 | 292.0 | 27.0 | 221.0 | 272.0 | 291.0 | 309.0 | 435.0 |
| 7 |  | North Central | 3287.0 | 495.0 | 34.0 | 234.0 | 471.0 | 495.0 | 517.0 | 642.0 |
| 8 |  | North East | 3287.0 | 648.0 | 42.0 | 255.0 | 620.0 | 648.0 | 676.0 | 826.0 |
| 9 | London | North West | 3287.0 | 751.0 | 54.0 | 379.0 | 713.0 | 752.0 | 788.0 | 1018.0 |
| 10 |  | South East | 3287.0 | 601.0 | 39.0 | 305.0 | 575.0 | 601.0 | 627.0 | 778.0 |
| 11 |  | South West | 3287.0 | 449.0 | 34.0 | 259.0 | 425.0 | 448.0 | 472.0 | 606.0 |
| 12 |  | ABL | 1982.0 | 526.0 | 35.0 | 331.0 | 503.0 | 526.0 | 550.0 | 691.0 |
| 13 |  | CKW | 1982.0 | 364.0 | 27.0 | 192.0 | 345.0 | 363.0 | 381.0 | 465.0 |
| 14 | York | Humb and ER | 1982.0 | 234.0 | 19.0 | 173.0 | 222.0 | 234.0 | 246.0 | 316.0 |
| 15 |  | North Yorks | 1982.0 | 272.0 | 25.0 | 196.0 | 255.0 | 272.0 | 288.0 | 390.0 |
| 16 |  | South | 1982.0 | 547.0 | 34.0 | 440.0 | 524.0 | 547.0 | 569.0 | 683.0 |
| 17 |  | Control Central and West | 1188.0 | 332.0 | 22.0 | 269.0 | 317.0 | 331.0 | 346.0 | 424.0 |
| 18 | Wales | Control North | 1188.0 | 260.0 | 19.0 | 199.0 | 247.0 | 260.0 | 273.0 | 341.0 |
| 19 |  | Control South East | 1188.0 | 411.0 | 30.0 | 323.0 | 392.0 | 412.0 | 432.0 | 585.0 |

Table S6: Comparison of number of calls across time series used in external validation
